# The Effect of Melatonin and Exercise on Social Isolation-Related Behavioral Changes in Aged Rats

**DOI:** 10.3389/fnagi.2022.828965

**Published:** 2022-02-08

**Authors:** Badrah Saeed Alghamdi

**Affiliations:** ^1^Department of Physiology, Faculty of Medicine, King Abdulaziz University, Jeddah, Saudi Arabia; ^2^Pre-Clinical Research Unit, King Fahd Medical Research Center, King Abdulaziz University, Jeddah, Saudi Arabia

**Keywords:** social isolation (SI), aging, melatonin, exercise, spatial memory, anxiety

## Abstract

Social isolation (SI) is well established as an environmental factor that negatively influences different behavioral parameters, including cognitive function, anxiety, and social interaction, depending on the age of isolation. Aging is a physiological process that is associated with changes in cognitive function, locomotor activity, anxiety and emotional responses. Few studies have investigated the effect of SI in senescence, or possible interventions. In the current study, we investigated the possible complementary effects of melatonin (MLT) and exercise (Ex) in improving SI-related behavioral changes in aged rats. Forty aged Wistar rats (24 months old) were randomly divided into five groups (*n* = 8 per group): Control (group housing), SI (individual housing for 7 weeks), SI + MLT (SI rats treated with 0.4 mg MLT/ml in drinking water), SI + Ex (SI rats treated with 60 min of swimming), and SI + MLT + Ex (SI rats treated with both MLT and Ex). Different behavioral tasks were conducted in the following sequence: open field test, elevated plus maze test, sucrose preference test, Y maze test, and Morris water maze test. Locomotor activities measured by total distance moved and velocity revealed that SI + Ex (*P* = 0.0038; *P* = 0.0015) and SI + MLT + Ex (*P* = 0.0001; *P* = 0.0003) significantly improved the locomotor activity compared with SI rats but SI + MLT (*P* = 0.0599; *P* = 0.0627) rats showed no significant change. Anxiety index score was significantly improved in SI + MLT + Ex (*P* = 0.0256) compared with SI rats while SI + MLT (*P* > 0.9999) and SI + Ex (*P* = 0.2943) rats showed no significant change. Moreover, latency to reach the platform in Morris water maze was significantly reduced at day 5 in SI + MLT + Ex (*P* = 0.0457) compared with SI rats but no change was detected in SI + MLT (*P* = 0.7314) or SI + Ex (*P* = 0.1676) groups. In conclusion, this study supports the possible potential of MLT in combination with Ex in improving physical activity, anxiety, and cognitive functions in aging population.

## Introduction

Aging is a normal physiological process and a known profounding factor for several disorders. The population growth rate in Middle East countries showed the highest rate compared with other countries over the last half century (Hajjar et al., 2013). Rapidly expansion of aging population raised many challenges and several concerns at national and international levels (Hajjar et al., 2013; Partridge et al., 2018). Alzheimer’s disease (AD) is one of the most known age-associated disease and a leading cause of dementia (Xia et al., 2018). According to AD association (2020), 10% of the American population age 65 and older has Alzheimer’s dementia (Jayaraj et al., 2020). In the United Arab Emirates, it is reported that AD affect one every seven people with age over 60 years old (Abyad, 2016). The risk of AD is exponentially rise with age to affect one every three people with age over 85 (Abyad, 2016; Jayaraj et al., 2020). Since then, geriatric research is expanding to reflect the importance of understanding the aging process and investigating some possible intervention of age-linked diseases. several studies have reported age-associated learning and memory impairment in different maze tests (Rapp et al., 1987; Gallagher et al., 1993; Shukitt-Hale et al., 2004; Robitsek et al., 2008; Mizoguchi et al., 2009) and high anxiety-related behavior in the open field and elevated plus maze (EPM) tests (File, 1990; Rowe et al., 1998; Darwish et al., 2001; Boguszewski and Zagrodzka, 2002; Bessa et al., 2005) compared with adulthood.

Social isolation (SI) is an environmental factor involving a lack of contact with members of the same species in the surrounding environment (Gong et al., 2017). It has been widely reported that SI negatively influences physical and mental well being (Cacioppo et al., 2011). In addition, previous studies have found that SI is associated with emotional and cognitive impairment (Serra et al., 2007; Fone and Porkess, 2008; McCormick et al., 2010). Moreover, SI is reported to accompany various brain disorders, such as schizophrenia (Jiang et al., 2013), bipolar disorder (Gilman et al., 2015), Alzheimer’s disease (AD) (Dong and Csernansky, 2009), and memory impairment (Steinbeck and Studer, 2015; Gong et al., 2017; Park H.-S. et al., 2020; Park S.-S. et al., 2020). It has been suggested that SI enhances stress hormones through activating the hypothalamic–pituitary–adrenal axis and mediating hyper-sensitivity to other stressors (Serra et al., 2005, 2007; Grippo et al., 2007).

Multiple studies have highlighted the important role of exercise (Ex) in physical and mental health during senescence (Callaghan, 2004; Sharma et al., 2006). Ex has been reported to improve anxiety, depression, mood disturbance, and social withdrawal (Guszkowska, 2004). A number of studies have confirmed a strong association between physical fitness and cognitive enhancement (Jedrziewski et al., 2007; Donnelly et al., 2016; Mandolesi et al., 2018; Lin et al., 2019). Moreover, it has been found that Ex enhances brain function through facilitating monoamine neurotransmitters (Daley, 2008). Ex plays a crucial role in hippocampal neuronal plasticity through regulating the neurotransmitter signaling pathway (Staples et al., 2015).

Melatonin (MLT) is a neurohormone secreted from the pineal gland and extra-pineal sources that exhibits a wide variety of regulatory functions (Alghamdi, 2018). One previous study reported that MLT enhanced memory by upregulating synapse-associated synaptophysin and postsynaptic density protein 95 genes in the prefrontal cortex (Alghamdi and AboTaleb, 2020). Other studies found that MLT can improve memory impairment though brain-derived neurotrophic factor/cAMP-response element binding protein expression in AD mice (Labban et al., 2021). MLT has also been shown to improve locomotor activity in a multiple sclerosis model through its potent anti-inflammatory and anti-oxidant effects (Abo Taleb and Alghamdi, 2020).

Some previous studies have shed light on the possible synergistic effects of MLT on Ex. This effective combination has shown a beneficial effect in a spinal cord injury animal model by enhancing neuronal stem cells proliferation (Park et al., 2010; Lee et al., 2014). Moreover, this combination improved locomotor activity through autophagy and apoptotic signaling pathways (Park et al., 2010). Other studies have suggested that MLT plus Ex can exert complementary effects in improving cognitive deficits and brain oxidative stress markers in 3xTg-AD mice (García-Mesa et al., 2012). These studies highlighted the potential additive effects of MLT and Ex in improving behavioral outcomes such as locomotor activity and cognitive function.

Although the effects of SI in early life and adulthood have been extensively studied (Ieraci et al., 2016; Locci et al., 2017; Liu et al., 2019; Screven and Dent, 2019), few studies have investigated this effect of SI in senescence (Shoji and Mizoguchi, 2011; Menec et al., 2020; Park H.-S. et al., 2020). In the current study, we hypothesized that MLT would have a complementary effect with Ex on SI-induced behavioral changes in aged rats.

## Materials and Methods

### Animals and Housing Conditions

A total of 40 male aged (24 months old, 500–600 g) Wistar rats were used. Male rats were used to avoid any possible hormonal effect on the experiment outcome from female rats. The rats were kept in a quiet, stress-free, temperature-controlled environment, on a 12-h light/dark cycle, with free access to water and food (chow). The current work was approved by the Biomedical Ethics Committee of King Abdulaziz University (Approval No. 03-CEGMR-Bioth-2021) and performed in the animal house at the King Fahd Medical Research Center.

### Treatment Groups

The rats were randomly distributed into five groups (eight rats per group). Group (I): Control group: group housing (GH) of two rats in large cages measuring 48 × 30 × 20 cm. Group (II): Individual housing (social isolation; SI) of rats in small cages measuring 20 cm × 26 cm × 13 cm for 7 weeks. Groups (III): SI and MLT in drinking water (SI + MLT). Group (IV): SI and exercise (SI + Ex). Group (V): SI with a combination of MLT and exercise (SI + MLT + Ex). Melatonin crystalline (M5250-10G) was dissolved in 100% ethanol and stored in aliquots at −70°C (Wolden-Hanson et al., 2000). Fresh drinking water consisting of 0.4 mg MLT/ml in a final concentration of 0.01% ethanol was prepared twice weekly. All drinking water bottles were covered with aluminum foil. Starting from week 2, Group III and V received MLT in their 0.01% ethanol drinking water (Wolden-Hanson et al., 2000). Group I, II, IV received only 0.01% ethanol drinking water.

### Body Weight and Food Consumption

Body weight and food consumption were measured at the beginning of each week from 08:00–9:00 a.m. The percentage of weight gain was calculated as: weight gain (%) = (new weight [W_1_] − initial weight [W_0_]/initial weight [W_0_]) × 100. The food consumption was measured per body weight (food intake [g]/body weight [g]) (Alghamdi, 2021).

### Swimming Exercise Protocol

Rats were familiarized with the swimming exercise at week 2. The familiarization stage started with 10 min of exercise on the first day and gradually increased to 60 min on the fifth day (De Sousa et al., 2020). Training was started on week 3 after 2 days of familiarization sessions. Swimming training sessions lasts for 60 min/day for 5 days/week and continued for 5 weeks (Ozturk and Ozdemir, 2020). Exercise sessions were conducted during the light cycle (from 09:00 a.m.–11:00 a.m.) (Lourenco et al., 2019). Rats swam separately in swimming tanks measuring (100 × 50 × 50 cm) containing tap water maintained at 24°C.

### Behavioral Tasks

**FIGURE 1:**
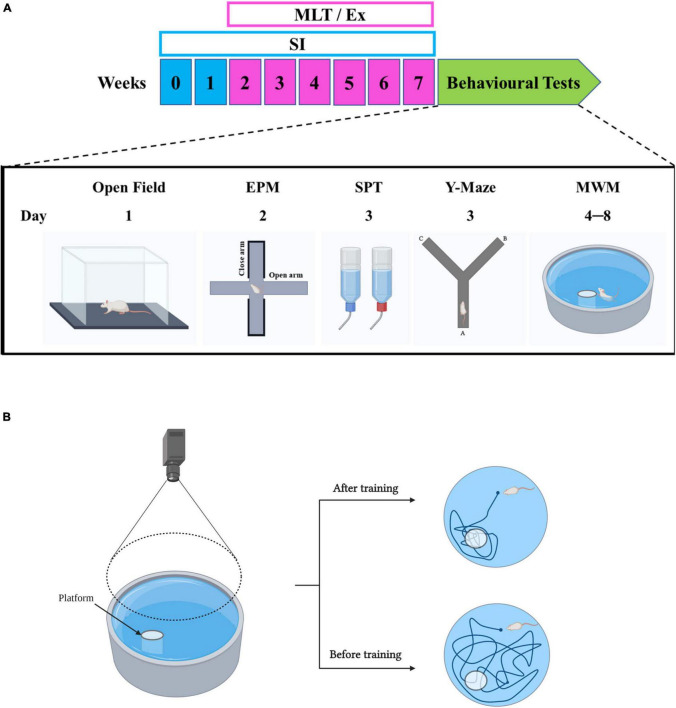
**(A)** Timeline of the experiment and overview of the behavioral tests. **(B)** MWM test. MLT, melatonin; Ex, exercise; SI, social isolation; EPM, elevated plus maze; SPT, sucrose preference test; MWM, Morris water maze. Created by BioRender.com.

Several behavioral tasks were conducted on week 8 in the sequence shown in Figure 1.

#### Open Field Test

The open field test was conducted in a square open arena and monitored with a EthoVision XT8A tracking system (Noldus Information Technology, Wageningen, Netherlands) for 3 min. The total distance moved (TDM), velocity, and immobility were automatically calculated by the tracking system. The frequency of rearing, in which the rats stood up on their hindlimbs, was recorded by the examiner. The arena was cleaned with 10% ethanol after each rat to reduce any odor bias. The tracking system calculated the time spent by the center point of the rat inside the central zone of the arena. The percentage of central zone preference was calculated as follows: Central preference% = (time in central zone/total experiment time) × 100.

#### Elevated Plus Maze

The EPM consisted of two closed and two open arms with a central zone. The rats were placed in the central zone and the duration and frequency of visiting each arm was automatically recorded with the ANY-maze version 6.23 (Stoelting Co., Wood Dale, IL, United States) tracking system for 5 min. The anxiety index was calculated as followed: anxiety index = 1 − ([open arm time/5 min] + [open arm entry/total entry])/2. Values closer to 1 indicate a higher level of anxiety (Dornellas et al., 2018).

#### Sucrose Preference Test

A two-bottle-choice test was used to measure the preference for 1% sucrose solution. Rats underwent 2 days of adaptation to two bottles of water (He et al., 2020). This was followed by a test in which rats were housed individually and could freely choose between the two bottles (bottle 1: 1% sucrose; bottle 2: water) for 12 h (He et al., 2020). In the middle of the experiment, the location of drinking bottles were switched. The consumption of sucrose is calculated as sucrose preference% = (sucrose consumption/total fluid consumption [water and sucrose]) × 100.

#### Y Maze

The Y maze consisted of three identical arms which met in the center at 120°. This test is a quick, simple and widely used test for assessing spatial memory in experimental animals. This type of memory can be assessed using the basic Y maze test to measure spontaneous alternation behavior. The rodent is placed in one arm and given the chance to freely move and explore all arms. Because of the rodent’s innate curiosity, they tend to explore previously unvisited arms (Lalonde, 2002). Consecutive entries into three different arms of the Y maze without repetition is known as spontaneous alternation behavior (Hughes, 2004). Entering a recently visited arm is considered an error which is reported in memory impaired experimental models. All four limbs of the rodent must be within the maze arm to constitute arm “entry.” The total number of arm entries is considered to reflect the activity state of rodents among groups, and is used to calculate the percentage of spontaneous alternation. The spontaneous alternation percentage (SA%) is calculated as follows: SA% = (number of alternations/[total number of arm entries − 2]) × 100, in which alternation is defined as consecutive entries into three different arms, such as abc, cba, bca, and bac.

#### Morris Water Maze

The MWM is a circular open pool (210 cm in diameter) half-filled with water, with a featureless interior surface. The MWM task is primarily designed to investigate long-term spatial learning and memory (Vorhees et al., 2000, 2004; Broening et al., 2001; Morford et al., 2002; Williams et al., 2003). The main concept of this task is to examine the ability of the rodent to use distal cues to navigate the pathway and locate a hidden platform despite different random starting points. In this task, the platform is placed in one quadrant and a semi-random set of starting positions is designed for daily learning trials (Figure 1B). The platform is camouflaged by adding opacifying dye to the water, such as tempera non-toxic paint to reduce the visual aspect ratio of the water, as seen by the animal when swimming. In cases of failure to reach the platform in 60 s, the rodent is guided to the goal and left on the platform for 15 s (Sutherland et al., 1987; Vorhees and Williams, 2006). To accurately collect all of the required parameters, the test sessions were recorded using ANY-maze version 6.23 (Stoelting Co., Wood Dale, IL, United States) tracking system software. Latency (s), the time spent from the start of the experiment to reach the platform, was recorded for all rats daily.

### Statistical Analysis

The data were analyzed using GraphPad Prism 8 (GraphPad Inc., La Jolla, CA, United States). Data are shown as mean ± standard error of the mean (Mean ± SEM). One-way analysis of variance (ANOVA) was conducted to analyze TDM, velocity, rearing, immobility, central preference, anxiety index, sucrose preference (%), SA%, and frequency of arm crossing in the Y maze. Repeated-measures ANOVA was conducted to analyze the% change in body mass, food consumption, and latency to reach the platform in the MWM. Tukey’s test was used as a *post hoc* test for significant ANOVA results, to compute confidence intervals for every comparison. Differences with *P* < 0.05 were considered to be statistically significant.

## Results

### Effect of Melatonin and Exercise on Body Weight and Food Consumption

**FIGURE 2:**
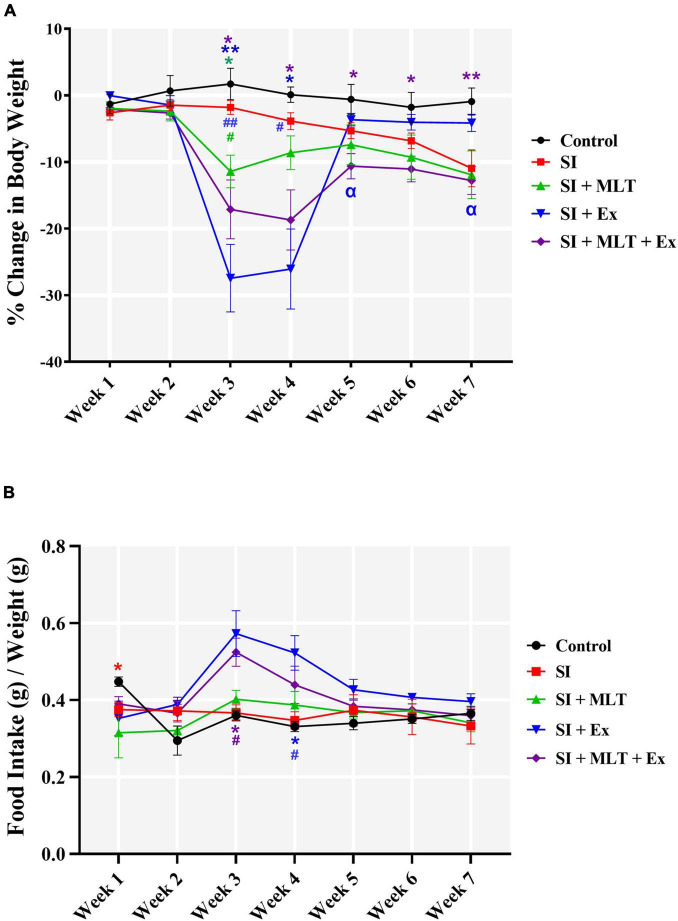
**(A)** The effect of MLT and Ex on the percentage change in body weight among groups of aged rats. **(B)** The effect of MLT and Ex on food consumption among groups of aged rats. Data are presented as mean ± standard error of the mean (SEM). Two-way repeated measures ANOVA was used, followed by Tukey’s multiple comparisons test. * indicates a significant difference between the treated groups and the control group at *p* > 0.05; # indicates a significant difference between the treated groups and the SI group at *p* > 0.05; α indicates a significant difference between the treated groups and the combination group at *p* > 0.05; **P < 0.01; ##*P* < 0.01. The color of the symbol denotes comparisons between groups with the matching color and line.

The effects of MLT and Ex on weight among groups were measured as the percentage of body weight change. Two-way repeated measures ANOVA showed a significant effect of weeks × treatment [*F*(28,243) = 7.683, *P*<0.0001], weeks [*F*(1.823,63.28) = 25.60, *P*<0.0001], and treatment [*F*(4,35) = 6.951, *P* = 0.0003], Figure 2A. Further analysis using Tukey’s *post hoc* test revealed that SI did not affect the percentage of weight gain compared with the Control group throughout the 7 weeks. However, MLT induced a decrease in weight gain on week 3 compared with the Control (*P* = 0.0124) and SI groups (*P* = 0.0321). Ex mediated the reduction in weight gain in week 3 compared with the Control (*P* = 0.0028) and SI groups (*P* = 0.0081), and in week 4 compared with the Control (*P* = 0.0189) and SI groups (*P* = 0.0429). A combination of MLT and Ex induced a reduction in weight gain in week 3 (*P* = 0.0222), week 4 (*P* = 0.0233), week 5 (*P* = 0.0298), week 6 (*P* = 0.0483), and week 7 (*P* = 0.0085) compared with the Control group. Moreover, a combination of MLT and Ex induced a reduction in weight gain in week 5 (*P* = 0.0475) and week 7 (*P* = 0.0295) compared with SI + Ex group.

The effects of MLT and Ex on food consumption were measured as the amount of food consumed during a full week. Two-way repeated measures ANOVA on food consumption showed a significant effect of weeks × treatment [*F*(24,184) = 2.116, *P* = 0.0030], weeks [*F*(3.827,117.4) = 5.857, *P* = 0.0003], and treatment [*F*(4,31) = 4.187, *P* = 0.0080] (Figure 2B). Further analysis using Tukey’s *post hoc* test revealed that the SI group exhibited reduced food consumption compared with the Control group on week 1 (*P* = 0.0479). However, Ex increased food consumption on week 4 compared with the Control (*P* = 0.0209) and SI groups (*P* = 0.0354). Moreover, combining MLT and Ex increased food consumption on week 3 compared with the Control (*P* = 0.0153) and SI groups (*P* = 0.0208).

### Effect of Melatonin and Exercise on Locomotor Activity

**FIGURE 3:**
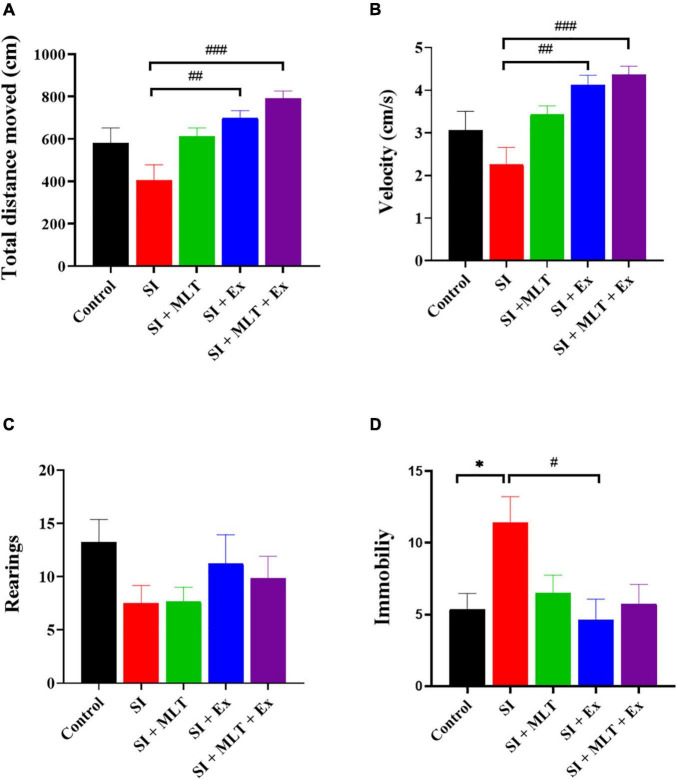
The effect of MLT and Ex in the open field test on **(A)** total distance moved, **(B)** velocity, **(C)** rearing frequency, and **(D)** immobility frequency among groups. Data are presented as the mean ± SEM. One-way ANOVA was used, followed by Tukey’s multiple comparisons test. * indicates a significant difference between the treated groups and the control group at *p* > 0.05; # indicates a significant difference between the treated groups and the SI group at *p* > 0.05; ##*P* < 0.01; ###*P* < 0.001.

There was a significant difference in TDM among groups [*F*(3,36) = 2.12, *P* = 0.0002; Figure 3A]. No significant change was detected in TDM between Control and SI (*P* = 0.1676). Also, no significant change was seen in the TDM between SI + MLT and SI group (*P* = 0.0599). The SI + Ex group showed higher TDM than SI group (*P* = 0.0038). SI + MLT + Ex group exhibited higher TDM than SI rats (*P* = 0.0001) but showed no significant change compared with SI + MLT (*P* = 0.1102) or SI + Ex (*P* = 0.7262).

There was a significant difference in velocity between groups [*F*(4,36) = 7.260, *P* = 0.0002; Figure 3B]. No significant change in velocity was detected between the Control and SI groups (*P* = 0.3966). Also, no significant change was seen in the velocity between SI + MLT and SI group (*P* = 0.0627). The SI + Ex group showed higher velocity than SI group (*P* = 0.0015). SI + MLT + Ex group exhibited higher velocity than SI rats (*P* = 0.0003) but showed no significant change compared with SI + MLT (*P* = 0.1758) or SI + Ex (*P* = 0.9811).

There was no significant difference in rearing frequency between study groups [*F*(4,37) = 1.464, *P* = 0.2329; Figure 3C]. No significant change was detected in the rearing frequency between Control and SI rats (*P* = 0.2901). Also, no significant change was seen in the rearing frequency in SI + MLT rats (*P* > 0.9999), SI + Ex (*P* = 0.6936), SI + MLT + Ex (*P* = 0.9125) compared with SI rats. Moreover, no significant change was detected in rearing frequency in SI + MLT rats (*P* = 0.9239) and SI + Ex (*P* = 0.9883) compared with SI + MLT + Ex rats.

There was a significant difference in immobility frequency between groups [*F*(4,34) = 3.531, *P* = 0.0163; Figure 3D]. SI rats exhibited significantly more immobility time compared with Control rats (*P* = 0.0362). No significant change was detected in immobility time in SI + MLT rats (*P* = 0.1250) compared with SI rats. SI + Ex rats exhibited significantly less immobility time compared with SI rats (*P* = 0.0143). No significant change was detected in immobility time in SI + MLT + Ex rats compared with SI (*P* = 0.0560), SI + MLT (*P* = 0.9950), and SI + Ex (*P* = 0.9771).

To sum up, SI + Ex and SI + MLT + Ex rats showed significantly higher TDM and velocity in comparison with SI rats while only SI + Ex rats exhibited significantly lower immobility time compared with SI rats.

### Effect of Melatonin and Exercise on Anxiety and Anhedonia

**FIGURE 4:**
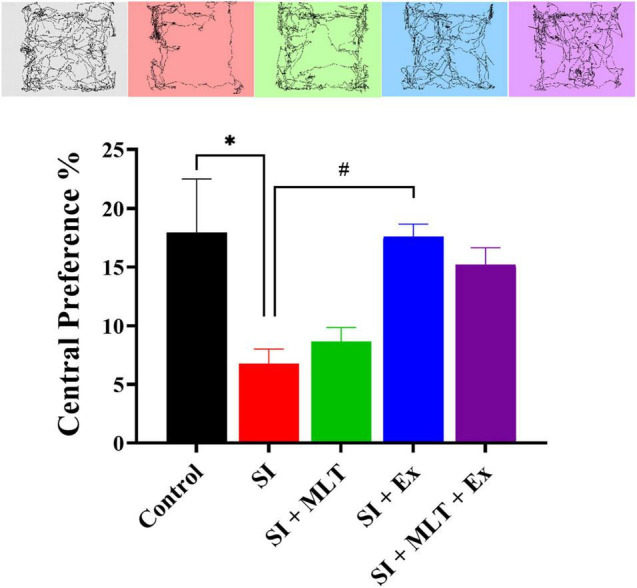
The effect of MLT and Ex on the central preference percentage among groups in the open field test. Data are presented as the mean ± SEM. One-way ANOVA was used, followed by Tukey’s multiple comparisons test. * indicates a significant difference between the treated groups and the control group at *p* > 0.05; # indicates a significant difference between the treated groups and the SI group at *p* > 0.05.

Regarding the central preference test in the open field test, there was a significant difference among groups [*F*(4,35) = 5.026, *P* = 0.0026; Figure 4]. There was a significant reduction in central preference in SI rats compared with Control rats (*P* = 0.0133). No significant change was detected in the central preference in SI + MLT rats (*P* = 0.9773) compared with SI rats. SI + Ex rats exhibited a significantly higher percentage of central preference compared with SI rats (*P* = 0.0174). No significant change was seen in central preference in SI + MLT + Ex rats compared with SI (*P* = 0.0970), SI + MLT (*P* = 0.2884), and SI + Ex (*P* = 0.9476) rats.

**FIGURE 5:**
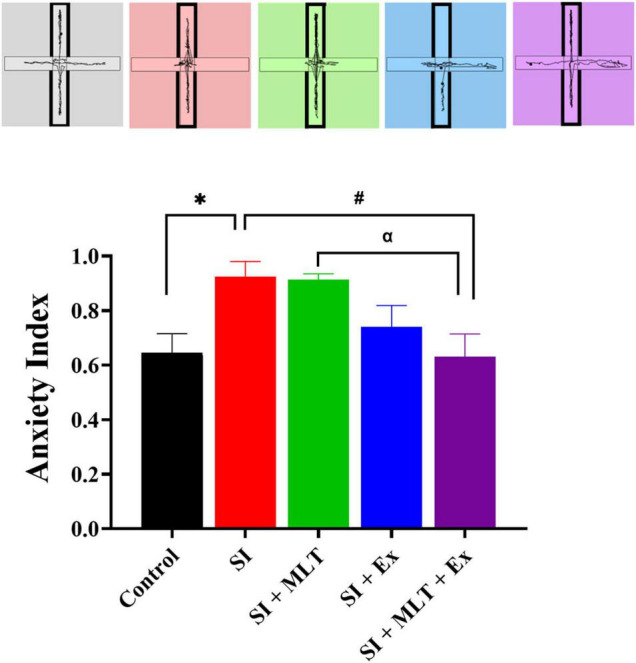
The effect of MLT and Ex on the anxiety index among groups in the EPM. Data are presented as mean ± SEM. One-way ANOVA was used, followed by Tukey’s multiple comparisons test. * indicates a significant difference between the treated groups and the control group at *p* > 0.05; # indicates a significant difference between the treated groups and the SI group at *p* > 0.05; α indicates a significant difference between the treated groups and the combination group at *p* > 0.05.

There was a significant difference in anxiety index scores among groups [*F*(4,35) = 4.599, *P* = 0.0043; Figure 5]. There was a significant increase in anxiety index scores in SI rats compared with Control rats (*P* = 0.0375). No significant change in the anxiety index in SI + MLT (*P* > 0.9999) and SI + Ex (*P* = 0.2943) compared to SI rats. SI + MLT + Ex rats exhibited significantly lower anxiety index scores compared with SI rats (*P* = 0.0256) and SI + MLT rats (*P* = 0.0359). No significant change was detected in anxiety index in SI + MLT + Ex compared with and SI + Ex (*P* = 0.7686).

**FIGURE 6:**
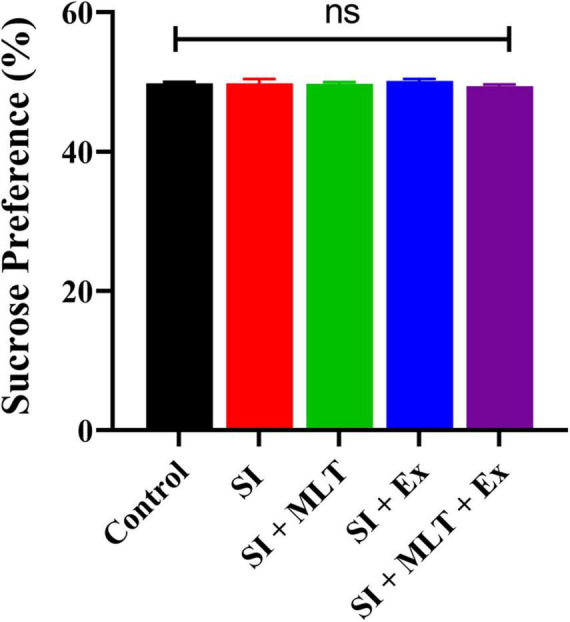
The effect of MLT and Ex on SPT performance (%) among groups. Data are presented as mean ± SEM. One-way ANOVA was used. ns, non-significant.

There was no significant difference in sucrose preference among groups [*F*(4,33) = 0.6693, *P* = 0.6179; Figure 6]. No significant change was detected in the sucrose preference between Control and SI rats (*P* > 0.999). Also, no significant change was seen in the sucrose preference in SI + MLT rats (*P* = 0.9993), SI + Ex (*P* = 0.9607), SI + MLT + Ex (*P* = 0.8988) compared with SI rats. Moreover, no significant change was detected in sucrose preference in SI + MLT rats (*P* = 0.9661) and SI + Ex (*P* = 0.4967) compared with SI + MLT + Ex rats.

To sum up, SI rats exhibited significantly lower central preference and higher anxiety index compared with Control group. SI + Ex significantly increased the central preference compared with SI group. Moreover, SI + MLT + Ex significantly decreased the anxiety index compared with both SI and SI + MLT groups.

### Effect of Melatonin and Exercise on Cognitive Function

**FIGURE 7:**
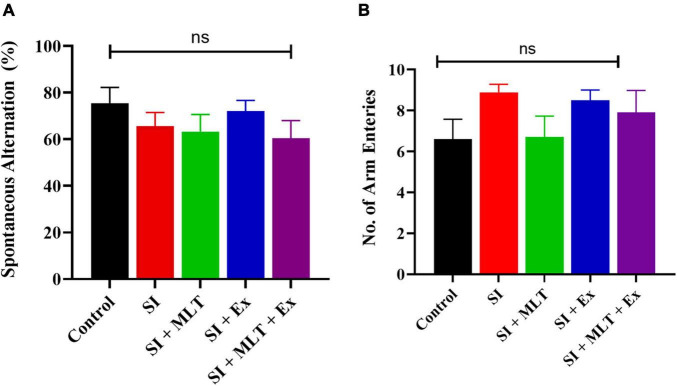
The effect of MLT and Ex on spontaneous alternation (%) **(A)**, and frequency of arm entries **(B)** among groups in the Y maze. Data are presented as the mean ± SEM. One-way ANOVA was used. ns, non-significant.

In the Y maze, there were no significant differences in spontaneous alternation (%) among groups [*F*(4,41) = 0.9599, *P* = 0.4397; Figure 7A]. No significant change was detected in the spontaneous alternation between Control and SI rats (*P* = 0.8090). Also, no significant change was seen in the spontaneous alternation in SI + MLT rats (*P* = 0.9991), SI + Ex (*P* = 0.9503), SI + MLT + Ex (*P* = 0.9796) compared with SI rats. Moreover, no significant change was detected in spontaneous alternation in SI + MLT rats (*P* = 0.9983) and SI + Ex (*P* = 0.6929) compared with SI + MLT + Ex rats.

Moreover, there was no significant difference in the number of arm entries among groups [*F*(4,44) = 1.445, *P* = 0.2352; Figure 7B]. No significant change was detected in the number of arm entries between Control and SI rats (*P* = 0.3554). Also, no significant change was seen in the number of arm entries in SI + MLT rats (*P* = 0.4004), SI + Ex (*P* = 0.9978), SI + MLT + Ex (*P* = 0.9286) compared with SI rats. Moreover, no significant change was detected in number of arm entries in SI + MLT rats (*P* = 0.8539) and SI + Ex (*P* = 0.9869) compared with SI + MLT + Ex rats.

**FIGURE 8:**
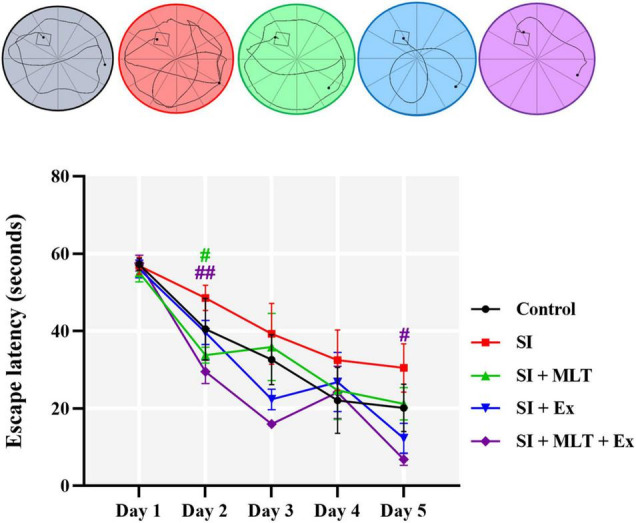
The effect of MLT and Ex on the latency to reach the platform in the MWM among groups of aged rats over 5 days of training. Data are presented as mean ± SEM. Two-way repeated measures ANOVA was used, followed by Tukey’s multiple comparisons test. # indicates a significant difference between treated groups and SI group at *p* > 0.05; ##*p* < 0.01. The color of the asterisks denotes comparisons between groups with the matching color and line.

In the MWM, two-way repeated measures ANOVA showed no significant effect of days × treatment [*F*(16,131) = 1.020, *P* = 0.4398]. However, the analysis revealed a significant effect of days [*F*(3.036,99.42) = 42.62, *P* < 0.0001], and treatment [*F*(4,34) = 3.473, *P* = 0.0175; Figure 8]. Further analysis using Tukey’s *post hoc* test revealed that the SI group did not affect the latency (s) to reach the platform compared with the Control group throughout the 5 days. However, MLT induced a reduction in latency on day 2 compared with the SI group (*P* = 0.0206). A combination of MLT and Ex induced a reduction in latency on day 2 (*P* = 0.0066) and day 5 (*P* = 0.0457) compared with the SI group.

To sum up, significant lower escape latency in MWM task was exhibited in SI + MLT rats at day 2 and SI + MLT + Ex rats at day 2 and 5 compared to SI rats.

## Discussion

The current study examined the possible complementary effects of MLT and exercise for attenuating SI-induced behavioral changes in aged rats. We subjected SI rats to moderate regular swimming exercise (60 min) and MLT. A series of behavioral tests was conducted to investigate the effects of a long period of SI (7 weeks) on aging and brain function in terms of locomotor function (TDM, velocity, rearing, immobility), anxiety (central preference% in open field test and anxiety index in EPM), depression (SPT) and cognitive function [Y maze (short-term spatial memory), and MWM (long-term spatial memory)].

Regarding weight and food consumption, our results revealed that at the end of 7 weeks of SI, there was no significant change in food consumption or weight compared with Control. This finding is consistent with a previous study reporting that neither 4 weeks nor 7 weeks of SI affected weight gain (Schiavone et al., 2016). Interestingly, we found that MLT administration ameliorated body weight changes, with no significant effect on food consumption. This result is consistent with a previous report that MLT administration in drinking water reduced the body weight of rats by 3% over 12 weeks with no significant change in food consumption (Wolden-Hanson et al., 2000). It is reported that endogenous MLT level is reduced with aging and thus alters metabolism and increases intraabdominal adiposity (Wolden-Hanson et al., 2000). Therefore, administration of exogenous MLT in aged rats can enhance metabolism and reduce weight independent of food intake. In addition, we found that exercise alone did not significantly affect body weight, which is consistent with a previous study reporting that short term (4 weeks) swimming exercise for 60 min did not affect the body weight of Wistar rats (Kim et al., 2011). The combination of MLT and Ex significantly reduced weight gain compared with the Control group. This is consistent with a previous study reporting that MLT + Ex significantly reduced weight on day 21 compared with a control group (Lee et al., 2014).

Different locomotor parameters were measured in the present study; TDM, velocity, rearing, and immobility. Our results indicated that SI did not significantly affect TDM, velocity or rearing behavior compared with the Control group. This is consistent with a previous study showing that different durations (2 and 4 weeks) of isolation did not significantly change the ambulation or rearing frequency in aged F344/N rats (Shoji and Mizoguchi, 2011). However, SI significantly increased the immobility frequency in our study compared with the Control group. A previous study reported that 4 weeks of SI ameliorated the immobility frequency of aged rats compared with group housed rats (Shoji and Mizoguchi, 2011). It should be noted that the duration of SI in our study was longer (7 weeks), which may have significantly reduced the immobility frequency. Exercise showed a beneficial effect on the locomotor activity of SI aged rats in terms of increasing TDM and velocity, and decreasing immobility frequency. This finding is consistent with previous studies reporting that exercise significantly reduced the immobility time in SI middle aged rats (Park H.-S. et al., 2020). Interestingly, a combination of MLT and Ex significantly improved TDM and velocity compared with both the SI. This finding is consistent with the use of this combination to enhance motor function in spinal cord injury animal models (Park et al., 2010).

Social isolation is known to negatively influence emotional and cognitive function. However, limited studies have investigated this effect in senescence. We tested anxiety using the open field and EPM tests, and tested anhedonia using the SPT. Our data revealed that SI for 7 weeks significantly affected anxiety levels in aged rats compared with Control rats. This was reflected in the significant reduction in central preference in the open field test and a significant increase in anxiety index scores in the EPM. These data are consistent with previous studies reporting a reduction in open arm entries in the SI group compared with the Control group in aged and adolescent rats (Park H.-S. et al., 2020; Park S.-S. et al., 2020). Exercise significantly improved the central preference percentage in aged SI rats compared with aged Control rats. This highlighted the importance of exercise in alleviating stress (Jiang et al., 2014). A combination of MLT and Ex significantly improved anxiety index scores of aged SI rats compared with aged Control rats. The SPT was used in the current study to investigate reward sensitivity behavior in which insensitivity to rewards is known as anhedonia, a sign of human depression (Liu et al., 2018). However, SPT performance exhibited no differences between groups. One potential explanation for this finding is that 7 weeks of SI was not sufficient to induce major depression measured by SPT, in accord with the absence of a significant change in weight or food intake between the Control and SI groups.

Social isolation has a negative influence on cognitive function, which can lead to deterioration in the presence of other risk factors, such as aging. In the current study, we focused on spatial memory; the Y maze and MWM were used to investigate short and long-term memory, respectively. On the one hand, the short-term spatial memory task indicated no significant change in SA among groups. On the other hand, long-term spatial memory measured using MWM escape latency exhibited amelioration in the SI group at day 5. This is consistent with previous studies reporting a negative impact of SI on cognitive function measured using the passive avoidance test, active avoidance, MWM, and novel object recognition test (Gong et al., 2017; Park H.-S. et al., 2020; Park S.-S. et al., 2020). In addition, we found that rats treated with MLT in combination with Ex exhibited significantly shorter escape latency compared with SI rats. Taken together with previous findings, the current results suggest that the combination of MLT + Ex could improve long-term spatial memory measured by MWM in aged SI rats.

Some studies have investigated the possible mechanisms of combined treatment of Ex Plus MLT. It has been reported that Ex plus MLT improved cognitive function and protected against brain oxidative stress in Alzheimer mice model (García-Mesa et al., 2012). Another study has reported that MLT + Ex significantly reduced level of inducible nitric oxide synthase mRNA which therefore decreased the secondary damage associated with spinal cord injury in rats (Park et al., 2010). Moreover, it has been shown that MLT in combination with Ex can increase proliferation of endogenous neural stem/progenitor cells and improved regeneration after spinal cord injury in rats (Lee et al., 2014).

## Conclusion

Previous research examining the effects of stress-induced behavioral changes in senescence is limited. The current results provided new insight regarding a possible complementary effect of combining MLT and Ex, which could potentially improve behavioral outcomes and quality of life in SI with aging. We investigated the possible effects of MLT and Ex in SI-induced behavioral changes in aged rats using different behavioral tasks. However, there are some limitations in the current study as small sample size, short-duration of interventions, and lack of histopathological and biochemical investigations. Moreover, different doses of MLT should be investigated to know the maximum synergetic effect that can be reached from this combination.

## Data Availability Statement

The data are available from the corresponding author upon reasonable request.

## Ethics Statement

This study was approved by the Ethics Committee of King Abdulaziz University (Approval No. 03-CEGMR-Bioth-2021) and followed the rules and regulations of the Animal Care and Use Committee (ACUC) at the King Fahd Medical Research Center (KFMRC), which comply with the guidelines of the “System of Ethics of Research on Living Creatures,” prepared by the King Abdulaziz City for Science and Technology, approved by Royal Decree No. M/59 on 24 August 2010.

## Author Contributions

BA did conception, experiment, data analysis and interpretation, and writing the article.

## Conflict of Interest

The author declares that the research was conducted in the absence of any commercial or financial relationships that could be construed as a potential conflict of interest.

## Publisher’s Note

All claims expressed in this article are solely those of the authors and do not necessarily represent those of their affiliated organizations, or those of the publisher, the editors and the reviewers. Any product that may be evaluated in this article, or claim that may be made by its manufacturer, is not guaranteed or endorsed by the publisher.
